# DnaK and DnaJ proteins from Hsp70/40 family are involved in Rubisco biosynthesis in *Synechocystis* sp. PCC6803 and sustain the enzyme assembly in a heterologous system

**DOI:** 10.1186/s12870-023-04121-1

**Published:** 2023-02-23

**Authors:** Małgorzata Rydzy, Piotr Kolesiński, Andrzej Szczepaniak, Joanna Grzyb

**Affiliations:** Department of Biophysics, Faculty of Biotechnology, University of Wrocław, F. Joliot-Curie 14a, Wrocław, Poland

**Keywords:** Rubisco, Chaperone, DnaK, DnaJ

## Abstract

Ribulose-1,5-bisphosphate carboxylase/oxygenase (Rubisco) catalyzes the first step of carbon fixation performed by photosynthetic organisms. Form I of this enzyme found in plants and cyanobacteria is composed of eight large (RbcL) and eight small (RbcS) subunits. To form a functional enzyme, Rubisco subunits need to be properly folded, with the assistance of cellular chaperone machinery, and consecutively assembled in a strictly orchestrated manner, with the help of multiple auxiliary factors. In recent years, multiple Rubisco assembly chaperones and their function in enzyme biogenesis have been extensively characterized. Little is known about the potential specialized factors involved in Rubisco subunits folding at the pre-chaperonin stage, yet this knowledge is greatly needed for the fast and efficient testing of new Rubisco variants.

*Synechococcus* sp. PCC 6803 Rubisco shows limited solubility and a lack of assembly in the *Escherichia coli* expression system. In this study, we aim to identify which additional chaperones are necessary and sufficient in sustaining the heterologous assembly of native Rubisco. Our findings prove that upon the introduction of *Synechocystis* DnaK2 to the *E. coli* system, RbcL is produced in soluble form. The addition of specific DnaJ (Sll1384) enhances this effect. We explain these combined effects based on binding constancies, measured for particular partners in vitro, as well as our analysis of the putative tertiary structure of the proteins. Our results have potential implications for Rubisco engineering.

**Supplementary Information:**

The online version contains supplementary material available at 10.1186/s12870-023-04121-1.

## Introduction

To sustain itself, the ecosystem needs a constant influx of inorganic carbon into organic matter. This function is maintained by photosynthetic organisms. A part of photosynthesis that is directly responsible for carbon fixation is called the Calvin-Benson-Bassham cycle [1]. The first step of the cycle is the CO_2_ incorporation into ribulose-1,5-bisphosphate (RuBP) by Ribulose-1,5-bisphosphate carboxylase/oxygenase (Rubisco). Besides the carboxylation reaction, Rubisco can fix oxygen to RuBP [2]. Due to its limited specificity and low catalytic rate, Rubisco is considered an imperfect enzyme. Therefore, it is not surprising that it has attracted the attention of scientists aiming to improve its properties [3, 4].

Research on Rubisco has been hampered for years by its complex biosynthesis pathway, preventing it from obtaining recombinant variants of this protein. Form I of this enzyme is composed of eight large (RbcL) and eight small subunits (RbcS), which assemble into a hexadecameric complex. To effectively build up such a holoenzyme, the presence of chaperonin and an assembly chaperone or assembly chaperone is required. It has been shown that the cooperation of bacterial GroEL/ES and either RbcX [5] or Raf1 [6] is necessary to obtain functional cyanobacterial enzymes in cell-free expression systems or heterologous expression in *Escherichia coli* cells. Biosynthesis of the Rubisco plant equivalents seems to be much more complex, and in *E. coli*, it involves the plant chaperonin Cpn60/Cpn20/Cpn10 and at least a few additional assembly chaperones, namely Raf1 and Bsd2 [7].

Although Rubisco folding in chaperonin and the consecutive assembly from its subunits have been extensively studied in recent years, little is known about the potential factors involved in the process at the pre-chaperonin stage. Here, we study the case of RbcL protein from *Synechocystis* sp. PCC6803 in the heterologous system of *E. coli*. It is assumed that this process may utilize the bacterial DnaK/DnaJ system. Being a member of the Hsp70/Hsp40 family, DnaK has already been shown as necessary for Rubisco folding during its heterologous expression [8]. DnaK is a 70-kDa heat shock response protein, with ATPase activity, involved in basic cellular processes such as the folding of newly synthesized proteins, preventing protein aggregation, protein transport and translocation, and regulative control of the proteins [9]. The lack of this protein might be lethal, as was shown for DnaK2 of Synechocystis PCC 6803 [10]. However, the sole presence of DnaK was insufficient for *Synechocystis* RbcL expression, indicating the need for other members of this chaperone family and their cooperation in the Rubisco assembly. This draws our attention to DnaJs, which are 40-kDa proteins with a highly conservative J-domain [11]. Most organisms pose multiple DnaJs.

*Synechocystis* sp. PCC6803 has seven DnaJs encoding genes. The resulting proteins are Sll1101, Sll1384, Sll0909, Sll1666, Sll0897, Slr0093, and Sll1933 (named after the Kazusa database nomenclature). One of these, Sll1933, appears to be especially crucial for *Synechocystis* sp. PCC6803 survival because the disruption of its gene is lethal. Little is known about the specificity of the particular DnaJs. Three of *Synechocystis* sp. DnaJs have additional domains in their structures. The Sll101 protein has the pentatricopeptide repeat (PPR**)** domain on its C-terminus, and the Sll1666 protein has a transmembrane region in its sequence, right behind the J domain. Sll1384 poses the tetratricopeptide repeat (TPR) domain on its C-terminus, which is a common motif involved inprotein–protein interaction [12, 13]. This protein was shown to be possibly engaged in phototaxis [14]. In general, specific interactions of DnaK proteins with defined DnaJs’ co-chaperones permit the control of the function and specificity of DnaK. In the presence of DnaJs, the ATPase activity of DnaKs is enhanced.

It has been assumed that cyanobacterial Rubisco is synthesized efficiently in *E. coli* cells if only an assembly chaperone is provided. In such a case, *E. coli* DnaK/DnaJ and GroEL/GroES machinery seem to substitute their cyanobacterial equivalents. However, a surprisingly small number of cyanobacterial proteins were obtained from the *E. coli* expression system (including those from *Synechocystis* sp. PCC 6301 [15], *Thermosynechococcus elongatus*, and *Synechococcus* sp. PCC 7002 [16]), contradicting the first claim. As previously reported [17], we have not been able to express Rubisco from *Synechocystis* sp. PCC 6803 in *E. coli*. Here, we decided to use this Rubisco as a test for the identification of potential chaperones used by cyanobacteria during RbcL folding at the pre-chaperonin stage. We aimed at all seven DnaJs of *Synechocystis* whose function is unclear, identifying one of them as the most probable RbcL binding protein and we quantified this binding by obtaining dissociation constants We also included DnaK2 as a possible partner of DnaJs, showing that DnaK is indispensable for RbcL biosynthesis. Therefore, we proved this in vivo, by analyzing Synechocystiss PCC 6803 knockout mutants, that the cooperation of DnaK and DnaJ is necessary for efficient biosynthesis of functional cyanobacterial Rubisco.

## Materials and methods

### Plasmid construction

Genes coding all seven DnaJs (*sll1101, sll1384, sll0909, slr0093, sll0897, sll1933,* and *sll1666*) and DnaK2 (*sll0707* gene) from *Synechocystis* sp. were amplified from the total DNA isolated from these cyanobacteria, using the primers listed in Table 1 and cloned (using restriction sites introduced with the primers) into pET16b plasmid (Novagen). The resulting constructs contained the polyhistidine tag (His-tag) encoding sequence on the N-terminus of cloned genes.

**Table 1:** List of the primers used for amplification of genes coding tested DnaJs. Restriction sites are underlined

Name of primer	Sequence
Fwsll1384	5’AAACATATG^NdeI^AGCTCGTTTCCCATCAAGCAAGG3’
Rvsll1384	5’AAAGAATTC^EcoRI^TTATTTCTTTTTGCCCCCGAATAACCC3’
Fw0909	5’AAACATATG^NdeI^GACGACCTCACCATCTACACAAGTCCG3’
Rv0909	5’AAAGAATTC^EcoRI^TTAAACCCCTAATTGTTCTTTTAAATCC3’
Fwsll0897	5’ AAACATATG^NdeI^TCATTTATGGAATAATCC3’
Rvsll0897	5’AAAGAATTC^EcoRI^ATGCCTGGGGATTATTACC3’
Fwslr0093	5’ AAACATATG^NdeI^GCATCAACAGATTTTCAAAG3’
Rvslr0093	5’AAAGAATTC^EcoRI^CTATGCCAACAAATTGGC3’
Fwsll1101	5’AAACATATG^NdeI^CAAGAGTTTTCCCACTATTACGAAATCCTGGG3’
Rvsll1101	5’AAAGAATTC^EcoRI^GGATTGATACGAATCACGGTAGCC3’
Fwsll1933	5’AAACATATG^NdeI^GAACAAGTGCGGAATTATTATCAAATTTTGG3’
Rvsll1933	5’AAAAAGCTT^HindIII^TTAAAAATCAAAGAACTTTTGCCG3’
Fwdnak2	5’ AAACATATG^NdeI^GGAAAAGTTGTTGGGATTG3’
Rvdnak2	5’AAAAAGCTT^HindIII^CTATTTCTCCGGCTCAGAG 3’

Plasmids for co-expression experiments (pUC18LXS, pCDFDnaK2, pACYCDnaJSll1384, and pET28aRaf1) were prepared using the NEB Gibson Assembly® kit (NEB) and following the manufacturer’s instructions.

### Protein expression

*E. coli* were grown using Luria‐Bertani (LB) broth or LB agar containing the following antibiotics when required: 100-μg·mL^−1^ ampicillin (Amp), 32-μg·mL^−1^ chloramphenicol (Cam), 100-μg·mL^−1^ spectinomycin (Spec), and/or 30-μg·mL^−1^ kanamycin (Kan).

DnaJs and DnaK2 were expressed in *E. coli* Lemo21. Expression media were inoculated with starter culture at a 5-ml:100-ml ratio and supplemented with 750-µM L- rhamnose. Bacteria were grown at 37 °C and 220 rpm until the culture reached an OD_600_ of ~ 0.7. Protein expression was induced by the addition of 0.7-mM IPTG, and the culture was continued at 25 ºC for 12 h. Cells were pelleted by centrifugation (15 min, 4,000 g, 4 °C), followed by further purification steps.

For co-expression of genes encoding Rubisco and tested chaperone proteins derived from the plasmids listed in Figure S5, *E. coli* BL21 Star™ (DE3) strain (Thermo Fisher Scientific Inc., Waltham, MA, USA) was used. Expression media were inoculated with starter culture at a 5- ml:100-ml ratio. Bacteria were grown at 37 °C and 220 rpm until the culture reached an OD_600_ of ~ 0.7. Protein expression was induced by the addition of 0.5-mM IPTG, and the culture was continued at 30 ºC for 10 h, when the culture was centrifuged (15 min, 4,000 g, 4 ºC). Pellets were kept frozen at -80 ºC.

### Cyanobacteria growth conditions

*Synechocystis* sp. PCC 6803 was cultivated on a BG-11 medium at 30 °C under continuous illumination of 50 µE × m^−2^ × s^−1^.

Antibiotics were added to the media for mutants of Δ*raf* [17] and Δ*sll1384* [12]: Kan (with a concentration of 100 µg/ml) and Cam (50 µg/ml). The Δ*raf1* was generated, as described in our previous work [17]; Δ*sll1384* was a kind gift from Professor Dirk Schneider.

### Protein purification

*E.coli* pellets were suspended in a 50-mM phosphate buffer (pH 6.5 for DnaJs and pH 7.5 for DnaK), with 150-mM NaCl, 1-mM PMSF, and 3-mM β-ME. Cells were disrupted by sonication and centrifuged (30,000 g, 30 min, 4 °C). The clarified lysate was then loaded on a Ni‐NTA resin (HisTrap 5 mL, GE Healthcare), equilibrated with a binding buffer (50-mM phosphate buffer, pH 6.5 for DnaJs, pH 7.5 for DnaK, 150-mM NaCl, 10-mM imidazole), and bound proteins were eluted using a gradient of imidazole (10–300 mM). Fractions containing DnaJ were pooled and dialyzed against the same buffer but without imidazole. Finally, the buffer was supplemented with 5% glycerol, and the protein was stored at -80 ºC. For DnaK, after the HisTrap step, fractions containing DnaK were pooled and loaded on a Superdex column 200 10/300, equilibrated with 50-mM phosphate buffer, pH 7.5, with 150-mM NaCl. Isocratic elution of proteins was done using the Akta Purifier system. Fractions containing pure DnaK2 were pooled, supplemented with 5% glycerol, and stored at -80 ºC.

### Protein isolation from cyanobacteria

After the cultivation, cyanobacterial cultures were pelleted by centrifugation (10 min, 3,000 g, 4 °C). Crude cell extracts were obtained by sonication (3 times, 1 min, at the highest power of the sonicator) in 20-mM Tris, pH 8.0, 150-mM NaCl, 2-mM PMSF, and 2-mM β-ME, followed by centrifugation (15 min, 15,000 g, 4 °C). The resulting supernatant was used for further experiments of co-migration of the complexes on the native gel and Rubisco activity assay).

### Isolation of RbcL from inclusion bodies

RbcL (originating from *Synechocystis* sp. PCC6803) was purified from inclusion bodies after expression (induced by 1 mM IPTG at 37 °C for 3 h) in *E. coli* BL21 (DE3). Harvested cells (30 min, 4500 g, 4 ºC) were resuspended in 40-mM Tris–HCl, pH 8.0, 1% (v/v) Triton X-100, supplemented with 1 mM PMSF and incubated on ice for 30 min, whereupon 0.5 M EDTA was added. Cells were disrupted by sonication, and inclusion bodies were pelleted by centrifugation (1 h, 22,000 g, 4 °C). Inclusion bodies were washed by resuspension in 40-mM Tris–HCl, pH 8.0, 10 mM EDTA, 1% Triton X-100, centrifuged (45 min, 30,000 g, 4 °C), and again homogenized with 40 mM Tris–HCl, pH 8.0, 10 mM EDTA, followed by centrifugation (45 min, 30,000 g, 4 °C). The pellet containing RbcL was solubilized in 40-mM Tris – HCl, pH 8.0, 4 M GdnHCl, 1 mM EDTA, and 5 mM β-ME. The protein concentration was set at 25 µM, and samples were stored at -80 °C.

### Displacement of 1,8-ANS assay

To monitor chaperone binding to the denatured unfolded RbcL peptide, the ANS assay was used. The 1,8-ANS is binding to hydrophobic surfaces on the proteins; therefore, folding or binding of a partner decreases the ANS load. Since ANS fluorescence is specific to the bound state, its replacement by chaperonins can be assessed by spectrofluorimetry. The fluorescence emission (470 nm) was monitored in kinetic mode, with a 390 nm excitation wavelength and 1 nm slits on both excitation and emission. Data were collected at 1 s intervals. Samples were continuously stirred and thermostated at 24 °C. For this experiment, RbcL was diluted to a final protein concentration of 10 µM with a 50-mM phosphate buffer, pH 7, and a final concentration of 0.5 M GndHCl. Next, ANS (from a 20 mM stock solution in water) was added to the sample, for a final 5 mM concentration, and incubated at 4 ºC overnight with constant mixing, for equilibration with a protein. For the measurement, the mixture was placed in a cuvette, thermostated at 22 °C. After recording the stable baseline of the fluorescence signal, the volume, corresponding to the desired concentration of a chaperone protein, was added. The measurement was followed until the changes reached a plateau. In this assay, all seven studied DnaJ homologs were tested (Sll1101, Sll1384, Sll0909, Slr0093, Sll0897, Sll1933, and Sll1666), as well as DnaK2 protein with ATP.

### Protein electrophoresis and immunoblotting

For native PAGE, a soluble fraction of *E. coli* culture, overexpressing the protein of interest, or a soluble fraction of cyanobacterial isolate was diluted with a 4 × native‐PAGE loading buffer (200-mM Tris‐HCl, 0.4% bromophenol blue, 40% glycerol). The volume, corresponding to a total of 20-μg protein, was loaded on BioRad TGX precast gradient native, 4–15% gel, 25 mM Tris‐HCl, and 190 mM glycine were used as running buffers. For SDS/PAGE, the soluble fractions of lysates or total lysates were diluted with a 4 × SDS/PAGE loading buffer [200 mM Tris‐HCl, pH 6.8, 0.4% bromophenol blue, 40% (v/v) glycerol, 8% (w/v) SDS]. The volume corresponding to 15 μg protein was loaded on a gel prepared by the von Jagov method [18]. The running buffers were 0.1 M Tris, 0.1 M Tricine, 0.1% SDS (a cathode), and 0.2 M Tris–HCl, pH 8 (an anode).

Immunoblotting was performed using the semi-dry transfer method on trans-blot BioRad. Proteins were transferred to a 0.45-μm Amersham™ Protran™ nitrocellulose or the PVDF blotting membrane (GE Healthcare) at 250 mA for 20 min, followed by blocking in 5% (w/v) skim milk powder in a TBS buffer (50 mM Tris‐HCl, pH 7.5, 150 mM NaCl) for 1 h before probing with a primary antibody (Table 2) for 45 min. The membranes were then washed thoroughly in a TBS-T buffer (50 mM Tris‐HCl, pH 7.5, 150 mM NaCl, 0.1% Tween-20), before probing with an anti‐rabbit IgG secondary antibody, HRP tagged (Sigma‐Aldrich, St. Louis, MO, USA) for 45 min in a TBS buffer. Subsequently, the membranes were washed 5 times for 1 min in a TBS-T buffer. The immunoblots were visualized using the chemiluminescence method. The HRP activity was assayed with freshly mixed luminol and enhancer: SuperSignal™ (Thermofisher Scientific) and detected with the BioRad ChemiDoc Imaging System.

**Table 2:** Antibodies used in this project and their references

Antibody	Catalog number	Supplier	Dilution
Anti-DnaK2	AS08 350	Agrisera AB	1:5,000
Anti-Raf1	custom production	Agrisera AB	1:2,500
Anti-RbcL	AS03 037	Agrisera AB	1:5,000
Anti-RbcS	AS07 259	Agrisera AB	1:5,000
Anti-RbcX	custom production	Agrisera AB	1:2,500
Anti-DnaJ Sll1384	custom production	Agrisera AB	1:2,500
Anti-His-tag HRP, conjugated	A7058	Sigmaaldrich	1:10,000
Goat anti-rabbit secondary antibody	AS09 602	Agrisera AB	1:10,000

### Microscale thermophoresis

To determine the K_d_ (dissociation equilibrium constant) of RbcL-DNAK2 or RbcL-DnaJ Sll1384 complexes, microscale thermophoresis (MST) was performed. For that, His-tagged proteins, namely DnaK2 or DnaJ Sll1384, were labeled with RED-tris-NTA dye (Nano Temper), following the supplier-suggested protocol. For that, 200 nM protein was incubated with 5 mM dye in 200 μl PBS-T buffer for 30 min a room temperature. For MST measurement, samples containing 4 μM or 200 nM of the labeled chaperone and unfolded RbcL (concentration ranges given in the results presenting figures) were prepared in a PBS-T buffer (phosphate-buffered saline, 0.1% Tween® 20) and loaded into a set of 16 capillaries (Monolith NT.115 MO-K022 capillaries, Nano Temper Technologies GmbH, Munich, Germany). The samples were analyzed with a Monolith NT.115 pico device under MO. Control Software (Nano Temper Technologies GmbH, München, Germany). Nano-red excitation with a LED light adjusted to 60% excitation power was used, while the infrared laser was set to high. The K_d_ value was calculated using the software’s built-in routines.

### Pull-down assay

To verify interactions between RbcL and the studied chaperones, Sll1384 and DnaK2, a pull-down assay was performed using His-tagged DnaK2 and Sll1384 as baits. A nickel resin was pre-equilibrated using a manufacturer protocol (cOmplete™ His-Tag Purification Resin). Next, 150 µl of a prepared mixture of the proteins of interest (50 µg of each protein) was added to 50 µl of resin and incubated for 30 min on a roller shaker with constant rotation. A resin was pulled down by centrifugation, and the supernatant was discarded. Next, potentially pulled-down proteins with their partners were washed from the resin with 400 mM imidazole. Controls had been done simultaneously; each protein used in the assay was incubated with a resin individually to exclude any unspecific binding of protein with resin. For this purpose, the versions of DnaK and DnaJ Sll1384 without a His-tag were prepared and tested.

### Rubisco activity assay

Rubisco carboxylase activity assay was performed, as previously described [16]. Simply, before the assay, the enzyme was incubated for 30 min, 30 °C in 50 mM Bicine–NaOH, pH 8.0, supplemented with 20 mM MgCl2, 2 mM DTT, and 20 mM NaH14CO2 (specific activity 1 μCi/μmol). The carboxylase reaction was started by the addition of RuBP (final concentration of 0.4 mM). The reaction was stopped at specific time points by the addition of 2 M HCl. After the samples drying, the residues were resuspended in 0.5 ml water, mixed with 4.5 ml of scintillation fluid, and quantified by liquid scintillation counting (Beckman LS-6500 scintillation counter). Specific activity was expressed as micromoles of 14CO2 fixed per minute per milligram of protein.

Equivalents of cyanobacterial cell extracts containing 20 μg of proteins were used for individual measurements.

### In silico analysis

A model of the DnaJ Sll1384 structure was generated using the SWISS-MODEL. The structure was analyzed using either Pymol or the SPDB viewer [19, 20]. Sequences were aligned using Clustal Omega [21]. A mood loop was used to model part of the DnaJ Sll1384 sequence [22]. Blastp was used to look for homological sequences of both DnaJ and DnaK among other organisms. Phylogenetic tree was prepared with the PACA bioinfo platform, available at phylogeny.fr [23].

## Results

### Sll1384 and DnaK2 from *Synechocystis sp*. PCC6803 interacts with unfolded RbcL in vitro

#### ANS displacement assay

**Fig. 1:**
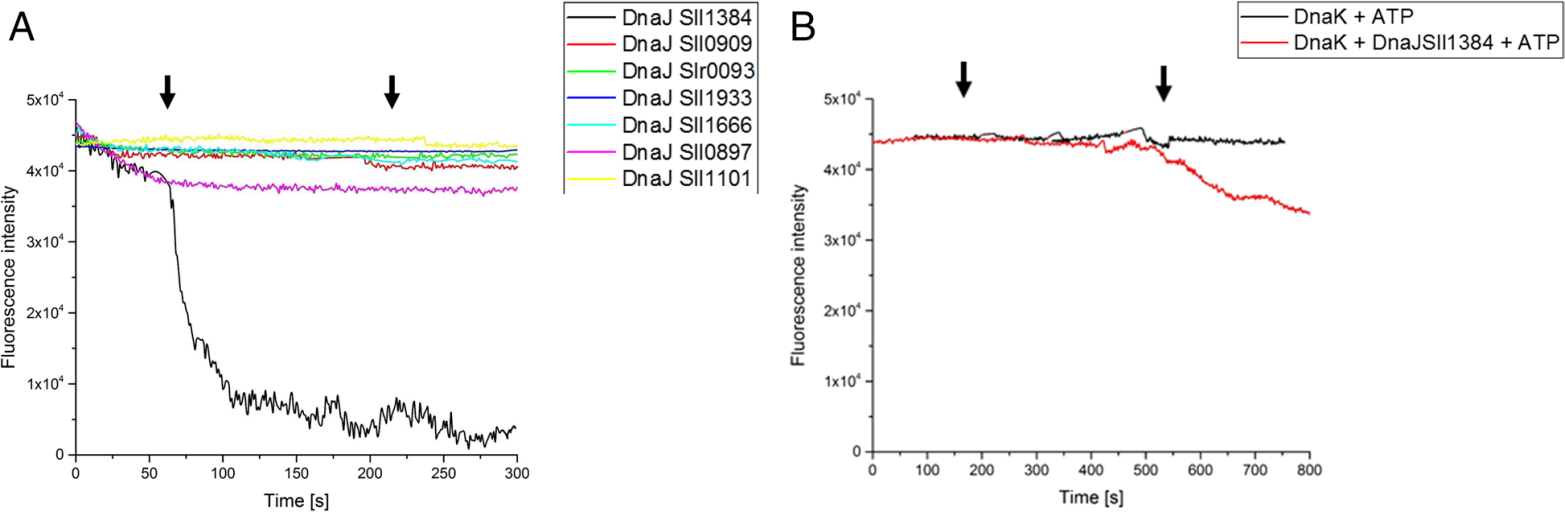
Binding of unfolded RbcL by DnaJs (**A**) or DnaK (**B**) from *Synechocystis* sp. PCC 6803. Displacement of 1.8 ANS (20 mM) from hydrophobic surfaces of unfolded RbcL (25 µM) upon binding of the chaperone was measured as a decay of fluorescence intensity at 470 nm (excitation 390 nm) within a 600–800-s time scale. Arrows indicate time points of a chaperone addition (final concentration of 5 µM). Plots represent three independent measurements. ATP was added to a starting mixture (at a final concentration of 5 mM) when DnaK2 was tested

To verify if any of the DnaJ proteins from *Synechocystis* sp. might directly bind RbcL, we used an ANS displacement assay. Examples of traces, recorded for this experiment, are shown in Fig. 1A. We observed a significant decrease in ANS fluorescence intensity only for Sll1384, indicating binding-related ANS removal from RbcL. Therefore, only this chaperone was identified as interacting with Rubisco. Therefore, DnaJ Sll1384 was used in consecutive experiments, characterizing the binding in more detail. We additionally tested a lower concentration of DnaJ Sll1384. The rates, calculated for the ANS displacement, are presented in Table S1. Interestingly, with a chaperone concentration decrease by about twice times, the reaction rate decreased more than ten times. A further drop in a chaperone amount did not significantly change the reaction rate. This point out to a chaperone concentration as a limiting factor in the Rubisco assembly process.

To further verify if DnaK2 from *Synechocystis* sp. PCC 6803 would bind to RbcL, and we performed an ANS displacement assay, as described for DnaJ representatives. We focused on DnaK2 only, as it is constitutively expressed and essential for cell survival in *Synechocystis* sp. PCC 6803. DnaK2 did not bind to RbcL in the presence of ATP alone, as no decrease in fluorescence was observed (Fig. 1B). This phenomenon is not unexpected since it was reported that not only ATP but also the presence of co-chaperone is needed for DnaK activity [10]. Therefore, we tested DnaK2 cooperation with the previously identified DnaJ Sll1384. We found a decrease in fluorescence intensity (Fig. 1B), indicating weak binding of DnaK to RbcL. The rate of fluorescence decrease was significantly lower than those shown in the interaction between RbcL and DnaJ Sll1384. The presence of ATP did not change the pattern. Therefore, we tested if DnaK would somehow interfere with DnaJ Sll1384 binding (Fig. 1B). The rate of the fluorescence intensity decrease is about 20 times lower in comparison to the one recorded for DnaJ alone. The presence of ATP did not alter the DnaJ behavior (not shown), we can thus conclude that DnaJ and DnaK compete for the same binding places on the RbcL molecule, or the binding of the first causes steric hindrance to the binding of the second.

#### Microscale thermophoresis

**Fig. 2:**
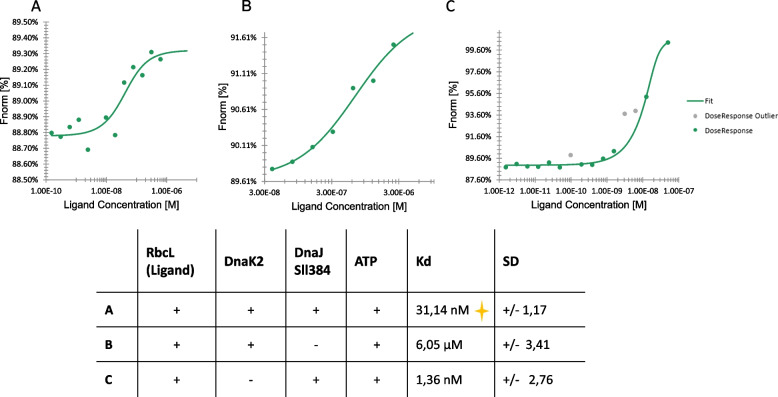
Microscale thermophoresis (MST) determination of dissociation constants characterizing the interaction of RbcL and its chaperone(s). Points are the results of the normalized fluorescence of individual capillaries while the bold line is a fit of the 1:1 binding model. Curves (A) RbcL + DnaK + DnaJ + ATP, (B) RbcL + DnaK2 + ATP, and (C) RbcL + DnaJSll 1384 + ATP are representatives of at least three independent repetitions. Experiments run for 4 μM (**A**, **B**) or 200 nM (**C**) of the labeled chaperone; unfolded RbcL concentration indicated at X axis, as ligand concentration. The table at the bottom of the figure summarizes K_d_s being the mean of all repetitions. The orange plus sign indicates the strongest binding

To quantify DnaK2 and DnaJ Sll1384 binding to the RbcL protein, we performed an MST experiment. For that, RbcL (200 nM) was titrated by variable amounts of DnaJ or DnaJ and DnaK2 in the presence of ATP. A titrant (DnaJ or DnaK2) was prelabeled by a red dye using Monolith His-Tag Labeling Kit RED-tris-NTA (NanoTemper Technologies) attaching to a His-tag. Examples of titration curves are shown in Fig. 2. We found that the weakest binder was DnaK2 (K_d_ = 6.05 ± 3.41 µM). Stronger binding was observed in the co-presence of DnaJ and ATP (K_d_ = 1.36 ± 2.76 µM). The lowest K_d_ (31.14 ± 1.17 nM) and the strongest binding were reported in the co-presence of both tested chaperones and ATP.

### DnaK and DnaJ proteins affect RbcL solubility in *E. coli* and co-migrate with Rubisco in native electrophoresis conditions

**Fig. 3:**
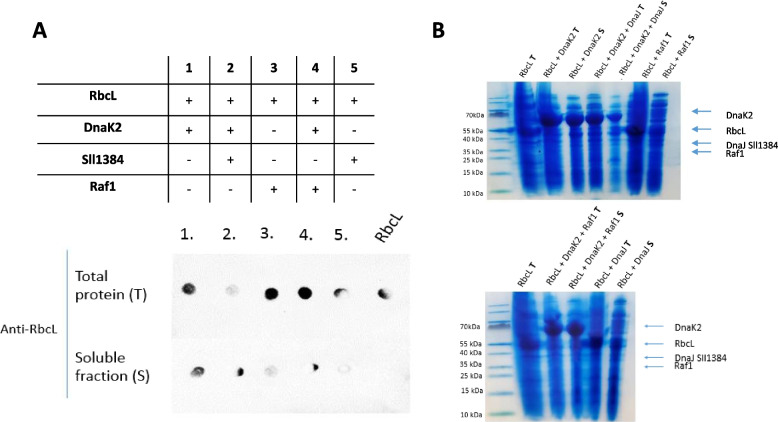
Effect of selected Rubisco chaperones on RbcL solubility in *E. coli* cells. **A** A table representing co-expression components and a dot blot (anti-RbcL antibodies) obtained for each (1–5) co-expression variant, indicating the presence of RbcL in a soluble fraction (S) and the total cell lysate (T). **B** A loading control (SDS-PAGE) to a dot-blot experiment, each line containing a volume of *E. coli* lysate corresponding to 20-μg total protein (T or S indicates a soluble or a total fraction). An expected migration of each factor is indicated with an arrow. The presented results are representative of three independent experiments

In our experiments, Rubisco from *Synechocystis* sp. PCC6803, even in the presence of RbcX, failed to be expressed in *E. coli* cells as a functional enzyme. RbcL was produced in great amounts but always accumulated in inclusion bodies. There is no available report of opposite results from any other laboratory. Therefore, having the in vitro evidence of DnaK and DnaJ binding to RbcL, we decided to challenge these chaperones in a heterologous expression system. We also co-expressed Raf1 since the complex of this protein with DnaK2 was already confirmed [17]. Our results show that only in the case of co-expression of RbcL with DnaK2, almost all of the RbcL goes to a soluble fraction (Fig. 3A, assay version 1). The same configuration of co-expression, with the addition of DnaJSll1384, enhances the solubilization of RbcL even more (, compare intensities of total and soluble fractions in version 2, Fig. 3A, B, and S8). Surprisingly, when RbcL is co-expressed with DnaK2 and Raf1, its amount in the soluble fraction decreases (Fig. 3A, assay version 4). Interestingly, the total amount of RbcL changed between experimental variants and did not correlate with the protein solubility. For well-soluble RbcL, the total amount of this protein is lower. This may point out to the mechanism of folding regulation—with proper chaperones, peptides might be released later, and therefore, total production might be lower due to available ribosome machinery.

### Co-expression of Rubisco coding genes with studied chaperone proteins

**Fig. 4:**
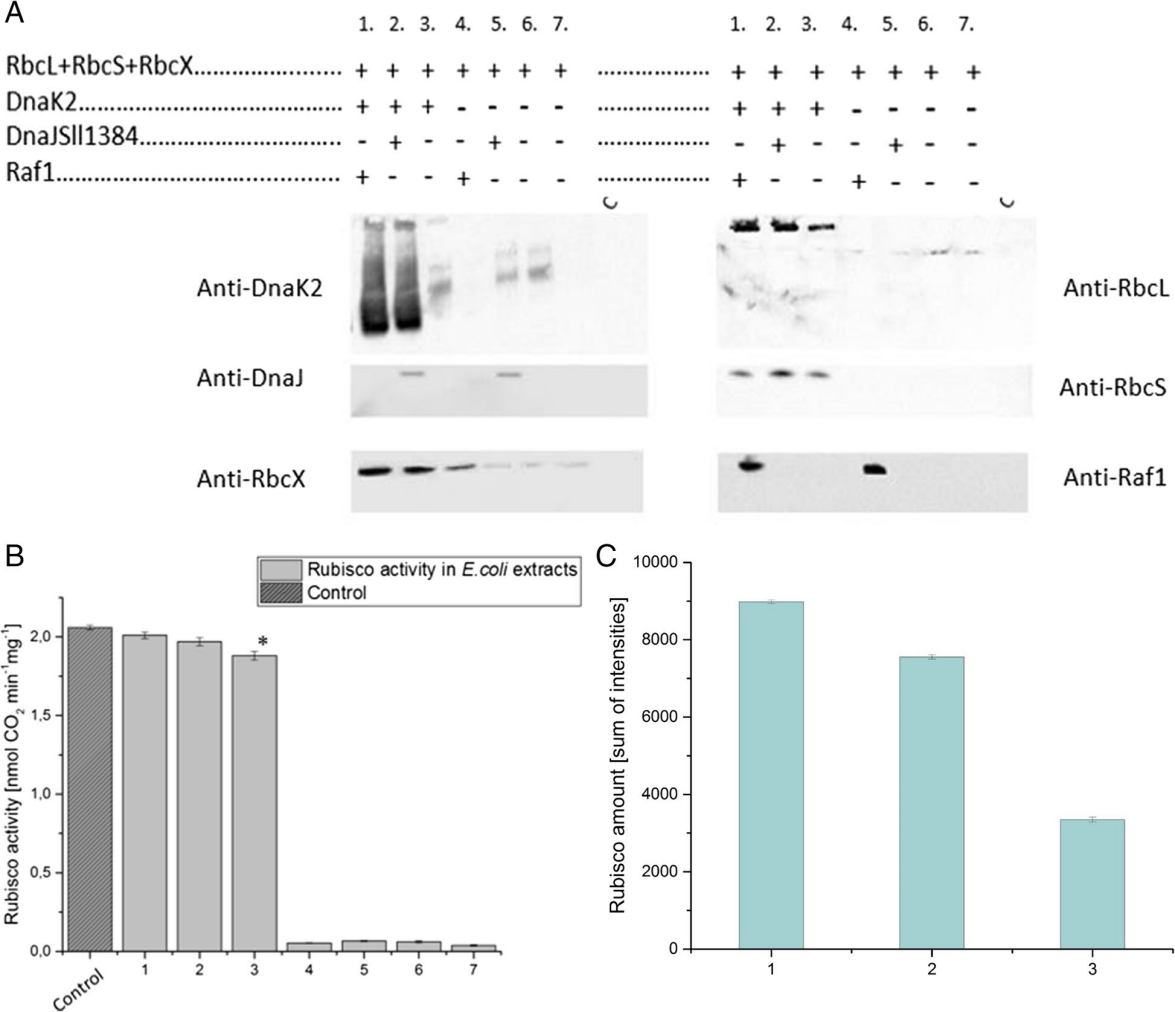
Results of co-expression of LXS operon from *Synechocystis* sp. PCC6803 with studied chaperone proteins. **A** Table showing a combination of genes used for co-expression (the mixture numbering, 1–7, is consequently used in the whole figure) and post-native PAGE western blots analyzed with antibodies anti: DnaK2, RbcL, DnaJ Sll1384, RbcX, RbcS, and Raf1. The last lane in the gel is an uninduced *E. coli* lysate to eliminate any possible antibodies background. Native PAGE samples were loaded based on equal OD600 (optical density of an *E.coli* sample measured at a wavelength of 600 nm) of cells. Original (full range) blots are available in supplementary, Figure S4. **B** Rubisco activity was measured in bacterial lysates obtained in particular co-expression combinations. The control (dark gray) is an expression of LXS operon from *Thermosynechococcus elongatus* in *E. coli* cells (see paragraph 3.3 for the rationale for this control). Statistical significance was determined using a one-way ANOVA with a Bonferroni posthoc test. *indicates the statistically significant difference (versus control) at *P* ≥ 0.05. **C** RbcL amounts in protein extracts after E.coli co-expression. The Rubisco amount was determined by densitometry of the Anti-RbcL signal. The bars present the means of the three measurements (± SD). Lanes are named 1,2,3 which corresponds to the number of co-expression setups presented in panel A

Knowing that DnaK and DnaJ Sll1384 increase RbcL solubility in a heterologous expression, we decided to test the interplay between a full set of structural Rubisco proteins (RbcL, RbcS) and RbcX, DnaK, DnaJ, and Raf1 in *E. coli* cells. For this purpose, we performed a co-expression of the LXS operon (coding RbcL, RbcX, and RbcS from *Synechocystis* sp. PCC6803) with the genes that encode the studied chaperone proteins: DnaK2, DnaJ Sll1384, and Raf1. The assembly of the Rubisco (RbcL + RbcS), as well as its activity, was then assayed. For the last case, Rubisco from *Thermosynechococcus elongatus*, assembling *in E. coli* without additional non-bacterial chaperones, was used as a reference. We obtained native (Fig. 4A) and active enzymes (Fig. 4B) in three co-expression configurations: LXS + DnaK2, LXS + DnaK2 + DnaJ Sll1384, and LXS + DnaK2 + Raf1. The amount of native enzyme slightly varied within co-expression variants, as indicated by densitometry analysis results (Fig. 4C). The native complex was composed of RbcL and RbcS. Additionally, the chaperones were bound strongly enough to stay with the complex during native electrophoresis.

### RbcL forms a complex with DnaK2 and DnaJ Sll1384 in vivo as confirmed by co-migration on native PAGE

**Fig. 5:**
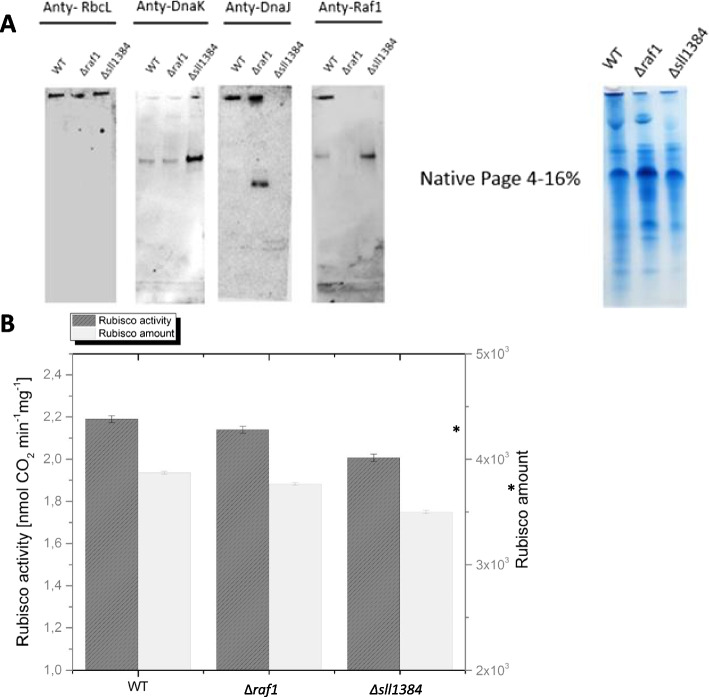
Rubisco and its chaperones in *Synechocystis* sp. PCC 6803 cells. **A** Soluble protein fractions from the wild-type *Synechocystis*, as well as its △Raf1 and △sll1384, were separated under native conditions and transferred to nitrocellulose for immune-blot analyses. The membranes were cut into four pieces and then probed with anti-RbcL, anti-DnaK, anti-DnaJ, and anti-Raf1 antibodies. The results shown here are representative, one of three independent experiments. **B** Comparison of RbcL amounts and Rubisco activity in protein extracts obtained from the listed *Synechocystis* variants. The Rubisco amount was determined by densitometry. The bars present the means of the three measurements (± SD). Statistical significance was determined using a one-way ANOVA with a Bonferroni posthoc test. *indicates the statistically significant difference (versus control) at *P* ≥ 0.05

The previous experiments confirmed that the tested chaperones interacted with the unfolded RbcL. These proteins were crucial for Rubisco holoenzyme assembly in a heterologous system. What remained to be confirmed, is the significance of these interactions in the native system of *Synechocystis* cells. Therefore, we separated a soluble fraction of lysate of Synechocystis using a native PAGE, and based onthis, we checked the co-migration of the RbcL with DnaK, DnaJ, and Raf1 (Fig. 5). We used wild type (WT), Δ*raf1* [24], and Δ*sll1384* strains of *Synechocystis,* as those secured the condition with all chaperones present (WT), no raf1 chaperone (Δ*raf1*) and no DnaJ chaperone (Δ*sll1384*). We did not test DnaK2 knockout, because this mutation is lethal [13]. The western blots indeed showed colocalization of bands, detected by anti-RbcL, anti-DnaK, anti-DnaJ, and Anti-Raf1 in WT cells (Fig. 5A). Moreover, DnaK2 always stayed with RbcL, even without additional chaperones (Fig. 5A, second gel). Interestingly, when the Raf1 chaperone was missing in the Δ*raf1* mutant, a quite high amount of free DnaJ Sll1384 was detected. It suggests that Raf1 somehow enhances the DnaJ affinity to RbcL or the DnaJ expression is increased to compensate for the lack of Raf1. Both hypotheses might be true since the intensity of DnaJ bands in Δ*raf1* mutants would suggest a higher expression of this chaperone, and in the Δ*sll1384* mutant, only a free (unbound) Raf1 protein was detected (Fig. 5A, fourth gel). The necessity of DnaJ for the correct Rubisco assembly was highlighted additionally by the comparison of the significantly lower activity of this enzyme in the Δ*sll1384* mutant (Fig. 5B). The lower activity correlated with the lower amount of soluble Rubisco.

We also checked if the RbcL peptide would be necessary for the interaction between particular chaperones, with the pull-down assay that had been done using the His-tag batch resin, where the His-tag was either on DnaK2 or DnaJ Sll1384 protein (Supplementary Figure S1). We confirmed the DnaK2:Raf1 and DnaK2:DnaJSll1384 complex, while as expected, DnaJSll1384 did not bind directly to the Raf1 protein. The presence of the RbcL complex did not change this pattern.

### Insights into the structure of studied DnaK2 and DnaJSlll1384

**Fig. 6:**
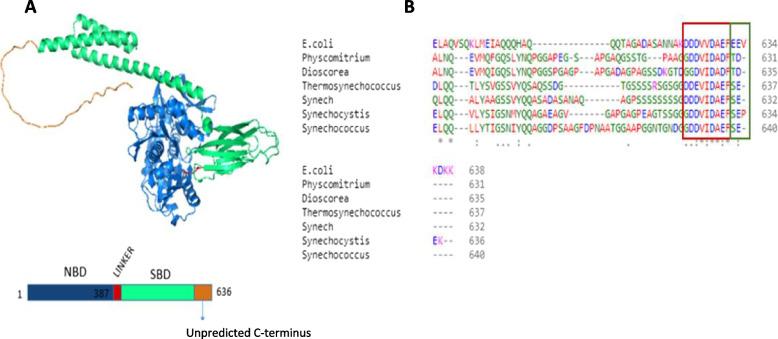
Structure analysis of DnaK2 from *Synechocystis* sp. PCC6803. **A** An AlphaFold model of DnaK2 and the schematic representation (rectangle below) of protein sequence, colored by domain: blue – nucleotide-binding domain (NBD), green – substrate-binding domain (SBD), orange – C-terminal tail. **B** Clustal Omega alignment of DnaK amino acid sequences from *E. coli* and its six cyanobacterial homologs. This figure presents only a part of the DnaK sequence (585–638) because this sequence is the most diverse. The whole sequence alignment is shown in the supplementary material (Figure S2)

The confirmation of the complex formation of DnaK2/DnaJ/RbcL leads to the question of the actual stoichiometry and binding places. To answer this question, it would be ideal to refer to the crystal structures of the complex, or at least, the chaperones. Unfortunately, there is no protein data bank (PDB) deposited structure of DnaK2. However, there is the AlphaFold [25] model with high-structure confidence (Fig. 6A). Based on this, the DnaK2 structure consists of the nucleotide-binding domain (NBD), the substrate-binding domain (SBD), and the C-terminal unstructured region. This is consistent with the DnaK2 of *E. coli.* However, the C-terminus of DnaK2 seems to be a key in substrate recognition, as it varies between *E. coli* and cyanobacterial proteins. The alignment (Fig. 6B) of six cyanobacterial DnaK homologs with *E. coli* DnaK reveals a high degree of sequence conservation among the tested protein sequences but a comparatively low similarity in the C-terminal tail (positions 604–638). Residues from this region of *E. coli* DnaK (positions 624–633) have been reported to be involved in protein binding [11]. This region has the highest conservation among the Hsp70 family [24]. It has also been shown that the C-terminal tail of DnaK, following residues (position 603), is most likely highly disordered. Taking these all together, we may postulate that this C-terminal part of DnaK2 is also responsible for RbcL peptide recognition. The *E. coli* DnaK protein also contains the glutamic acid-glutamic acid-valine (EEV) peptide directly behind the conserved motif (positions 624–633) [24]. Surprisingly, the position in the cyanobacterial DnaK that is supposed to correspond to the EEV sequence from *E. coli* differs and consists of TD/SE amino acids (Fig. 6B). This position seems to be conserved among cyanobacterial DnaKs and thus might have some specific yet unknown function.

**Fig. 7:**
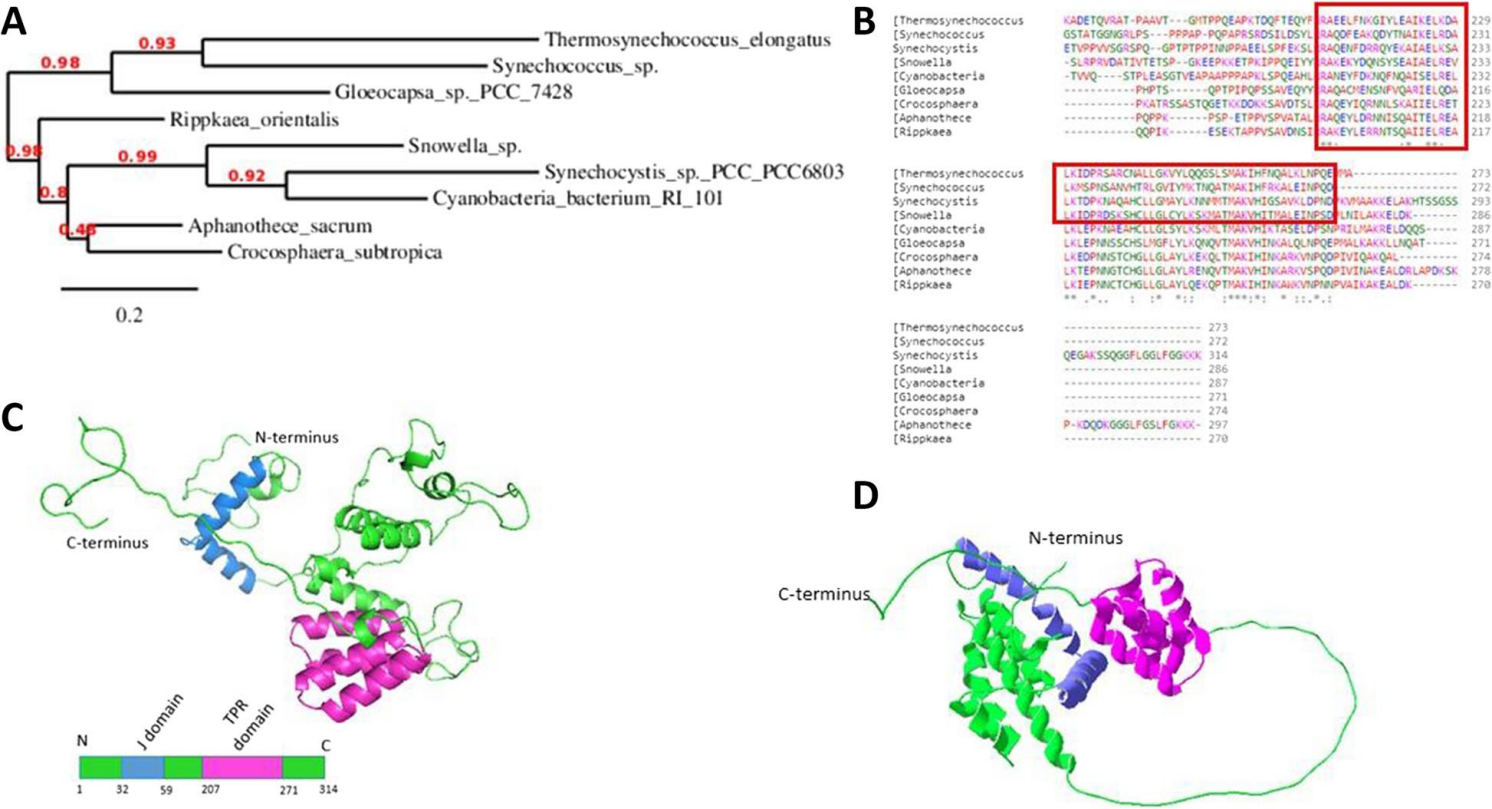
Model of DnaJ Sll384 structure obtained in this study*.* **A** Phylogenetic tree of the TPR domain, based on an alignment of DnaJ Sll1384 sequences from different cyanobacteria (**B**); the TPR domain is marked with the red frame. The alignment was made with Clustal Omega. The whole sequence alignment is shown in the supplement (Figure S3). **C** A ribbon representation of the 3D model of DnaJ Sll384 and a block scheme showing the color code used in the figure and **D** respective AlphaFold model of DnaJ Sll1384, colored by the same scheme

DnaJ Sll1384 of *Synechocystis* sp. PCC6803 is less related to DnaJs of *Thermosynechococcus elongatus* or *Synechococcus* sp. (Fig. 7A and B) than those chaperones are related to each other. Interestingly, the Rubiscos of both mentioned cyanobacteria can be easily expressed in *E. coli* cells [16, 26]. This indicates that during evolution, some small but crucial changes occurred, which forced the addition of another chaperone into Rubisco biosynthesis.

There is no crystal structure of Sll1384 in the PDBTherefore, we modeled DnaJ Sll1384 using the SWISS-MODEL tool. As there is no crystal structure of proteins that shares a high degree of homology with the whole DnaJ Sll1384, some parts of the model were missing in the SWISS-MODEL. The regions with low confidence were modeled next by using PyMol [27, 28], and the energies of the outcome structure were minimized by using the Modloop tool [22]. The final model is presented in Fig. 7C. It shows the typical feature of Hsp40, the N-terminal J domain with the conserved histidine-proline-aspartic acid (HPD) motif. The TPR domain, placed at the C-terminus of protein, consists of four α-helices.

The DnaJs’ loop region (helix II and helix III junction) shows a characteristic HPD motif. This motif is conservative and necessary for stimulating the ATPase activity of DnaK proteins. During the paper review process, an Alpha Fold model for DnaJ Sll1384 appeared (Fig. 7D). The model still has regions with low confidence, especially the long fragment connecting TPR and J domains. The TPR domain is present in both models as a compact, barrel-like helix coil. The characteristic J-domain helices, with an HPD motif, are also present in the Alpha Fold model, although the rest of the domain seems to be more structured than in our computation. All this together suggests that the chaperone has flexible regions, most probably dedicated to interaction with targets.

Homologs of DnaJ Sll1384 from other cyanobacteria also possess the TPR domain, with certain conservative residues that are typical of TPR domains. These are alanines in positions 8, 20, and 27, with the exceptions of *Crocosphaera*, *Snowella*, and *Cyanobacterium* in position 27, and prolines in position 32, with the exception of the *Synechococcus* sequence [29]. DnaJ Sll1384 has a unique TPR domain in its sequence, which is absent in the remaining six DnaJs of *Synechocystis*. The rest of the sequence of all cyanobacterial DnaJ Sll1384 shares a high level of similarity. The only region that precedes the TPR sequence seems to differ in each aligned sequence, meaning that it might be the place responsible for the specific RbcL peptide recognition.

## Discussion

Understanding Rubisco biosynthesis demands a detailed description of its chaperones. As RbcL with high sequence homology is expressed with different levels of success in a heterologous system of *E. coli*, it is reasonable to conclude that different chaperones are needed. Going further, we may claim that the chaperones’ specificity to particular RbcLs is high, and they may not substitute for each other. It is already known that some Hsp70/40 family members are involved in cyanobacterial Rubisco biosynthesis [8] but without clear identification. Here, we show that to obtain soluble, active Rubisco of *Synechocystis* sp. PCC6803 in E. coli, we also need to add DnaK2, DnaJ Sll384, and Raf1 chaperones. We have proven that the same system functions in vivo.

We quantified the binding of DnaJ and DnaK2 to denatured RbcL peptides. DnaJ and DnaK separately were binding with K_d_ at the micromolar scale. This finding is consistent with K_d_s known for DnaKs and their substrates (e.g., 0.2–1 μM [30] for interactions with short peptides, 1.52 μM for luciferase [31]). When both DnaJ and DnaK were present, K_d_ decreased by two orders of magnitude, showing the strong cooperation of those chaperones. Synergistic binding of those chaperones is expected [32]; however, at first sight, this seems to contradict the binding rates measured by our ANS displacement assay. There, we find that in the presence of DnaK and ATP, the binding of DnaJ is about 20 times slower. To explain this, we need to remember what measures MST (namely the dissociation equilibrium constant) and the ANS assay (minute removal of ANS). A decrease of the latter may simply mean that fewer molecules of ANS are removed from RbcL. It may result from less available space, as the binding of DnaK causes special hindrance. It may also indicate the lowering of the DnaJ binding–unbinding cycle (note that in our experiment, the DnaJ concentration was five times lower than its substrate concentration). This observation offers us additional insights into the mechanism of cooperation, suggesting that fast folding with just one chaperone probably leads to an erratic structure. Therefore, the unfolded peptide is stabilized with a multimeric chaperone complex. Such folding takes longer but results in a better product. The complex of DnaK/DnaJ may be preformed before binding to RbcL, as we detected it by a pull-down assay without a Rubisco template present.

**Fig. 8:**
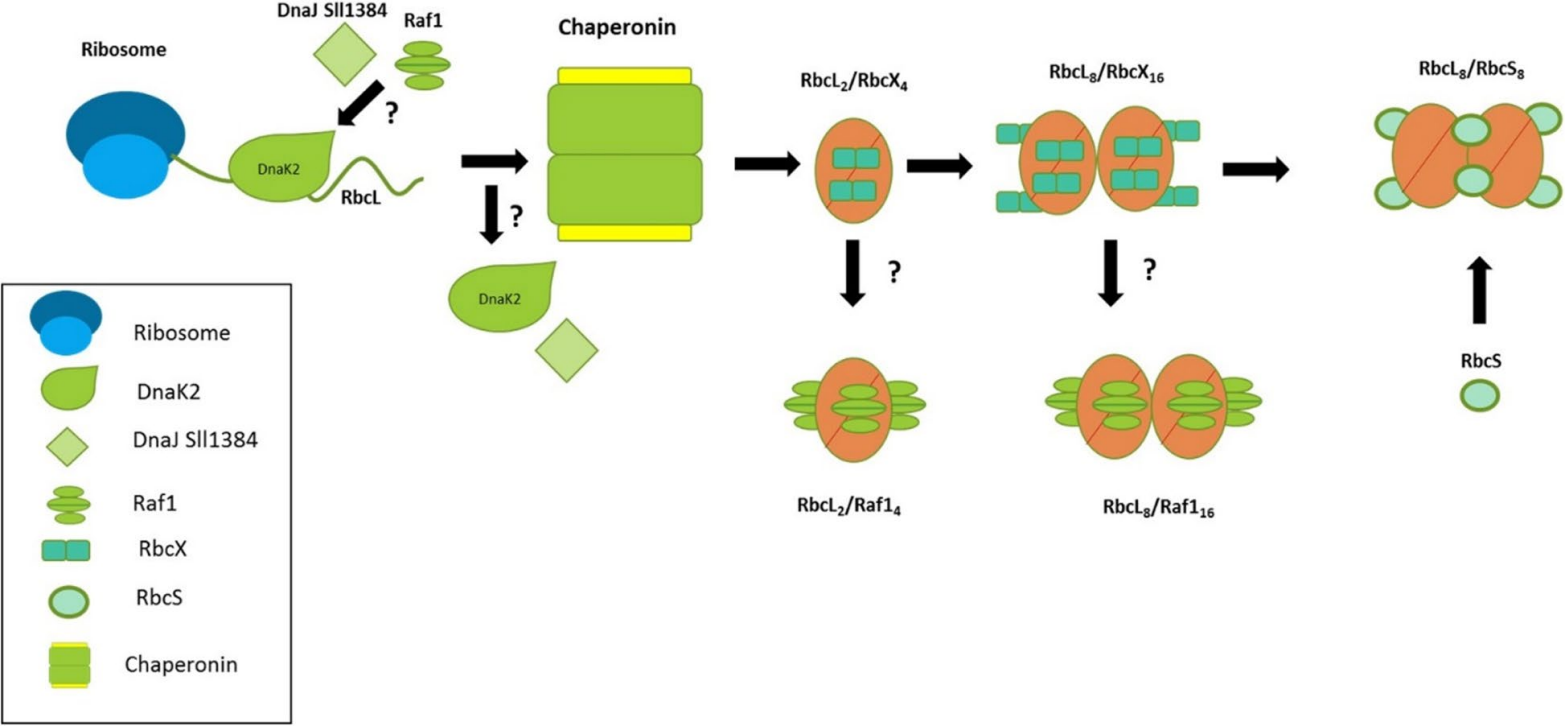
Model of biosynthesis process of Rubisco from Synechocystis sp. PCC6803. Specific proteins, participating in the process, are shown by their ideograms, described in a legend. Unclear points are indicated with question marks. Shortly, unfolded RbCL peptide, after the translation stage, bound to DnaK2, DnaJ SII1384, and/or Raf1. RbcL is then delivered to the chaperonin complex. Somewhere at this stage DnaK2 and DnaJ dissociate and the RbcX chaperone attaches. In the following steps, dimerization and further oligomerization of RbCL occur and RbcS connects, to form a functional Rubisco enzyme. The possibility of Raf1 substitution for RbcX in RbCl dimer/octamer is shown as an alternative pathway

We may understand the necessity of chaperones by looking deeper into the Rubisco folding process. Due to this, there is a high likelihood of misfolding by an unspecific interaction. Multimerization of RbcL is driven by hydrophobic interactions; there is a strongly hydrophobic contact surface between the RbcL monomers in the dimer. There is also a high number of labile structural elements, while the mutual orientation of the RbcL monomers is assured only by two symmetrically arranged salt bridges. Without the participation of additional factors, a non-functional intermediate form emerges [33]. This mechanism is summarized in Fig. 8.

*Synechocystis* sp. PCC6803 has seven DnaJs, while only DnaJ Sll1384 takes part in RbcL folding. The answer to the specificity of this DnaJ must therefore be in its amino acid sequence. The most significant difference is the presence of the TPR domain in DnaJ Sll1384. There are 23 proteins in *Synechocystis* sp., found with the TPR domain. Among these, 12 have been described to be involved in the photosynthesis process. For example, Ycf3 is a chaperone of the photosystem I (PSI) complex formation [34]. In *Chlamydomonas reinharditi*, an MRL1 protein with a TPR domain regulates the accumulation of the chloroplast *rbcL* gene transcript by stabilizing the mRNA via its 5′ UTR [35]. Here, we showed for the first time that DnaJ Sll1384 may indeed influence Rubisco biosynthesis on the level of protein folding and assembly. The TPR domain of DnaJ might be involved in this process. The Δ*sll1384* mutant of *Synechocystis* sp. PCC6803 has not been identified as the one with the disrupted Rubisco folding. The main feature identified was a very weak movement toward the light [14]. It is unclear how this is correlated with the novel function of this protein as a chaperone in Rubisco biosynthesis. Both functions might be independent, or the phototaxis function may be activated by cell stress.

The requirement of DnaK2 in the heterologous expression of *Synechocystis* sp. RbcL in *E. coli* shows that endogenous DnaK from *E. coli* is not fully sufficient here, as the co-presence of DnaJ increases Rubisco activity and solubility. DnaK2 of *Synechocystis* sp. and DnaK of *E. coli* share a high level of similarity, which might be enough for function substitution. The C-terminal region of DnaK2 has lower homology, drawing our attention to its feature as again a probable place of interaction with RbcL or DnaJ. It has been shown that this region is involved in substrate binding [24]. The *E. coli* DnaK protein and eukaryotic Hsp70 homologs both contain the EEV motif in this C-terminal region. This sequence is broadly believed to comprise TPR interaction plates [24]. Although there is no clear EEV motif in cyanobacterial DnaKs that is supposed to correspond to the EEV sequence, there is a conserved TD/SE motif that might have the same function, namely the interaction with the TPR domain of DnaJ. DnaJ proteins deliver substrate proteins to DnaK and accelerate the ATPase activity of the latter [36]. Therefore, we prove again that the binding of DnaJ to RbcL and DnaK2 to RbcL and the interaction between DnaJ and DnaK2 are both necessary.

Overall, our study presents the first report with confirmed participation of specialized and conserved cyanobacterial Hsp70/40 proteins, DnaK2, and DnaJ Sll1384 in Rubisco biosynthesis. The co-expression of those proteins with LXS operon in *E. coli* increases RbcL activity and yield. These are key findings, which allow further studies on Rubisco improvements and production in heterologous systems.

## Supplementary Information

**Additional file 1: Figure S1.** Pull-down of complexes formed by RbcL and its chaperones. (A) composition of mixtures, tested with a his-tag batch resin as baits, “+” represents presence of particular proteins (B) a dot-blot analysis (antibodies version indicated in a figure) of baits-bound proteins for particular mixtures and (C) a control SDS-PAGE of pull-downed proteins. M - a mass marker. Mixtures contained 50 μg of a given protein per total 150 μl of a mixture. Bound proteins were washed out with 400 mM imidazole. **Figure S2. **Clustal omega alignment of whole amino acid sequence of *E.coli* DnaK and its six cyanobacterial homologues. **Figure S3. **Clustal omega alignment of whole sequences of DnaJ Sll1384 from different cyanobacteria species. TPR domain marked with a red frame. **Figure S4.** Full range blots data for Fig 4. **Table S1.** Rate of fluorescence decrease, representing chaperone binding to RbcL peptide, measured by ANS displacement assay. DnaJ/DnaK (final concentration indicated) were added directly to RbcL-ANS mixture. **Figure S5.** Cloning cassette in plasmids used for co-expression, with indicated protein gene and specific resistance (amp- ampicillin, spc- spectomycin, chl- chloramphenicol, kan- kanamycin). Names of plasmid given in each rows. **Figure S6.** Kinetics of Rubisco activity. Panel A presents kinetics of Rubisco activity in cyanobacterial extracts (activity presented on the Fig 5B in the manuscript), panel B presents kinetic of Rubisco activity presented on the Fig 4B in the manuscript). **Figure S7.** Full blots for Fig 5. **Figure S8.** Full dot blots from Fig 3. **Figure S9.** Densitometry analysis of dot-blots of Fig 3. (A) Total RbcL and soluble RbcL quantification, bars are the average of results obtained for two independent biological replicates, error bars are the maximal deviation for that probe. (B) the relative content of soluble RbCl in its total fraction. Sample coding (1-5) as on Fig. S8.

## Data Availability

All data generated or analyzed during this study are included in this published article and its supplementary information files.
