# Supplementary material for: DnaK and DnaJ proteins from Hsp70/40 family are involved in Rubisco biosynthesis in Synechocystis sp. PCC6803 and sustain the enzyme assembly in a heterologous system
Source: BMC Plant Biol. 2023 Feb 23;23:109. doi: 10.1186/s12870-023-04121-1 (PMC9948308; doi:10.1186/s12870-023-04121-1)
Supplement: Supplementary file 1 — Additional file 1: Figure S1. Pull-down of complexes formed by RbcL and its chaperones. (A) composition of mixtures, tested with a his-tag batch resin as baits, “+” represents presence of particular proteins (B) a dot-blot analysis (antibodies version indicated in a figure) of baits-bound proteins for particular mixtures and (C) a control SDS-PAGE of pull-downed proteins. M - a mass marker. Mixtures contained 50 μg of a given protein per total 150 μl of a mixture. Bound proteins were washed out with 400 mM imidazole. Figure S2. Clustal omega alignment of whole amino acid sequence of E.coli DnaK and its six cyanobacterial homologues. Figure S3. Clustal omega alignment of whole sequences of DnaJ Sll1384 from different cyanobacteria species. TPR domain marked with a red frame. Figure S4. Full range blots data for Fig 4. Table S1. Rate of fluorescence decrease, representing chaperone binding to RbcL peptide, measured by ANS displacement assay. DnaJ/DnaK (final concentration indicated) were added directly to RbcL-ANS mixture. Figure S5. Cloning cassette in plasmids used for co-expression, with indicated protein gene and specific resistance (amp- ampicillin, spc- spectomycin, chl- chloramphenicol, kan- kanamycin). Names of plasmid given in each rows. Figure S6. Kinetics of Rubisco activity. Panel A presents kinetics of Rubisco activity in cyanobacterial extracts (activity presented on the Fig 5B in the manuscript), panel B presents kinetic of Rubisco activity presented on the Fig 4B in the manuscript). Figure S7. Full blots for Fig 5. Figure S8. Full dot blots from Fig 3. Figure S9. Densitometry analysis of dot-blots of Fig 3. (A) Total RbcL and soluble RbcL quantification, bars are the average of results obtained for two independent biological replicates, error bars are the maximal deviation for that probe. (B) the relative content of soluble RbCl in its total fraction. Sample coding (1-5) as on Fig. S8. [file 12870_2023_4121_MOESM1_ESM.docx]

**Supplementary material**

**DnaK and DnaJ proteins from Hsp70/40 family are involved in Rubisco biosynthesis in *Synechocystis* sp. PCC6803 and sustain the enzyme assembly in a heterologous system**

**Małgorzata Rydzy, Piotr Kolesiński, Andrzej Szczepaniak, Joanna Grzyb***

**Department of Biophysics, Faculty of Biotechnology, University of Wrocław**

**al. F. Joliot-Curie 14a, Wrocław, Poland**

***corresponding author: joanna.grzyb@uuwr.edu.pl**


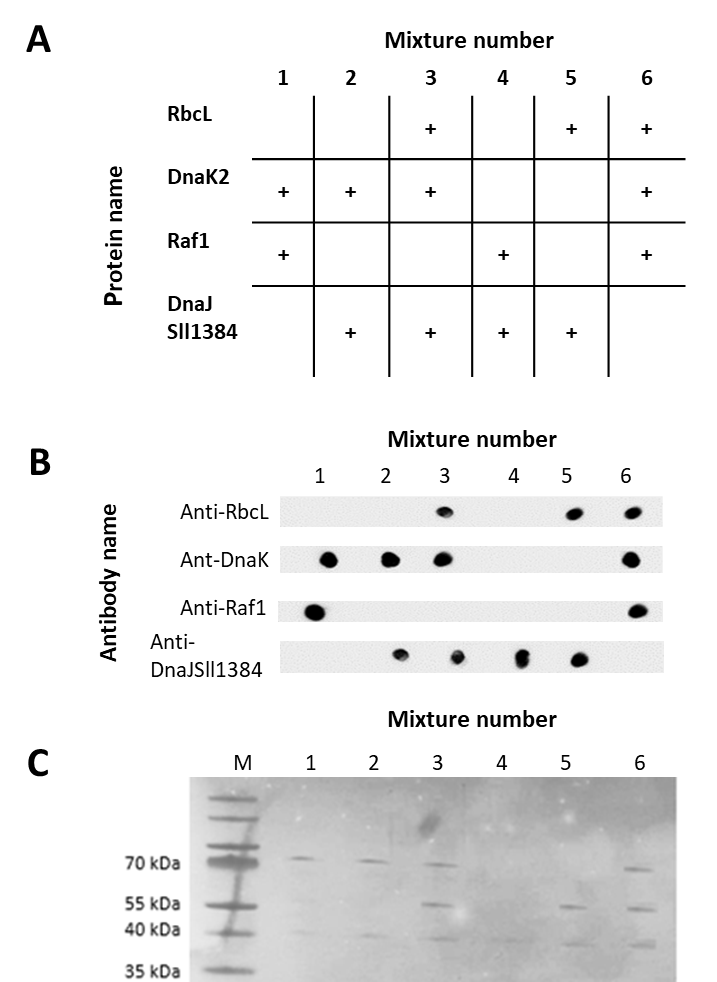


**Figure S1**. Pull-down of complexes formed by RbcL and its chaperones. (A) composition of mixtures, tested with a his-tag batch resin as baits, “+” represents presence of particular proteins (B) a dot-blot analysis (antibodies version indicated in a figure) of baits-bound proteins for particular mixtures and (C) a control SDS-PAGE of pull-downed proteins. M - a mass marker. Mixtures contained 50 μg of a given protein per total 150 μl of a mixture. Bound proteins were washed out with 400 mM imidazole.

**
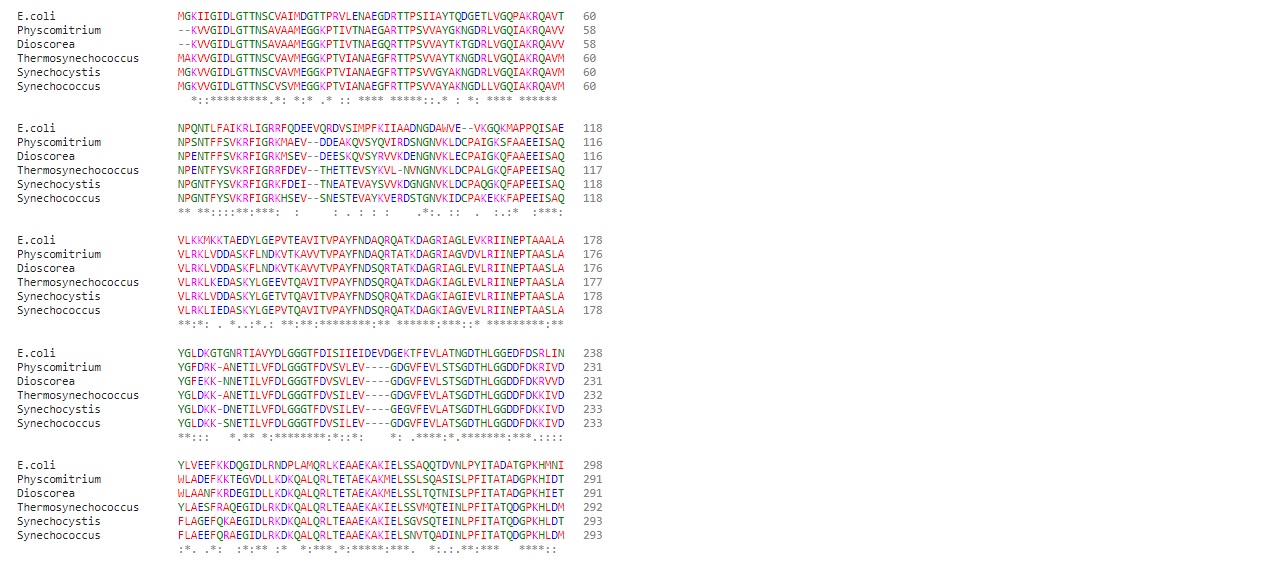
**

**
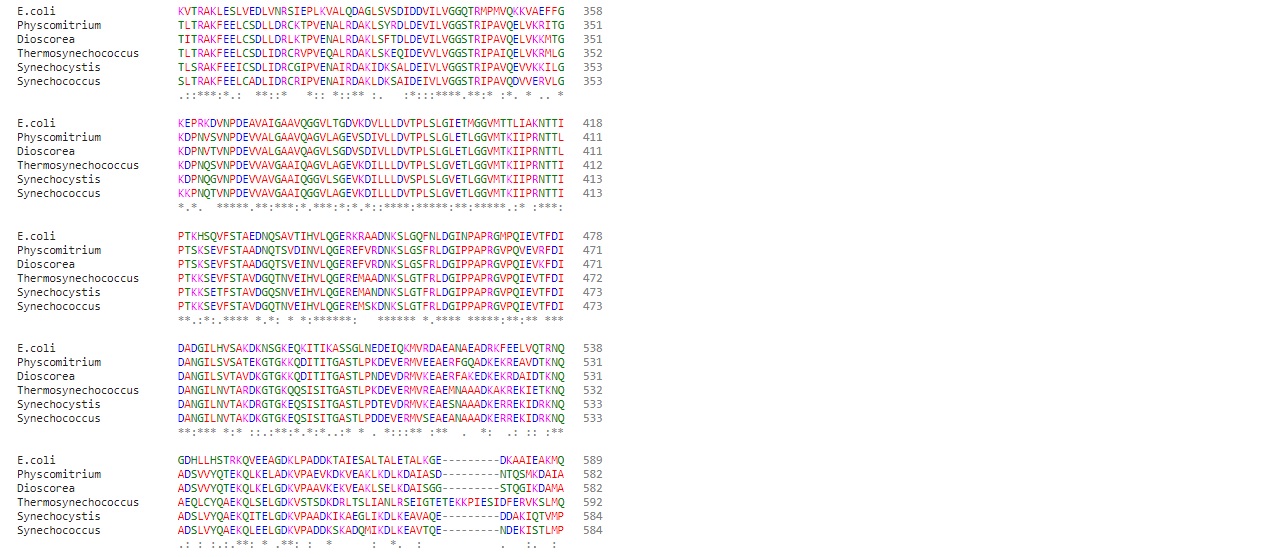
**

**
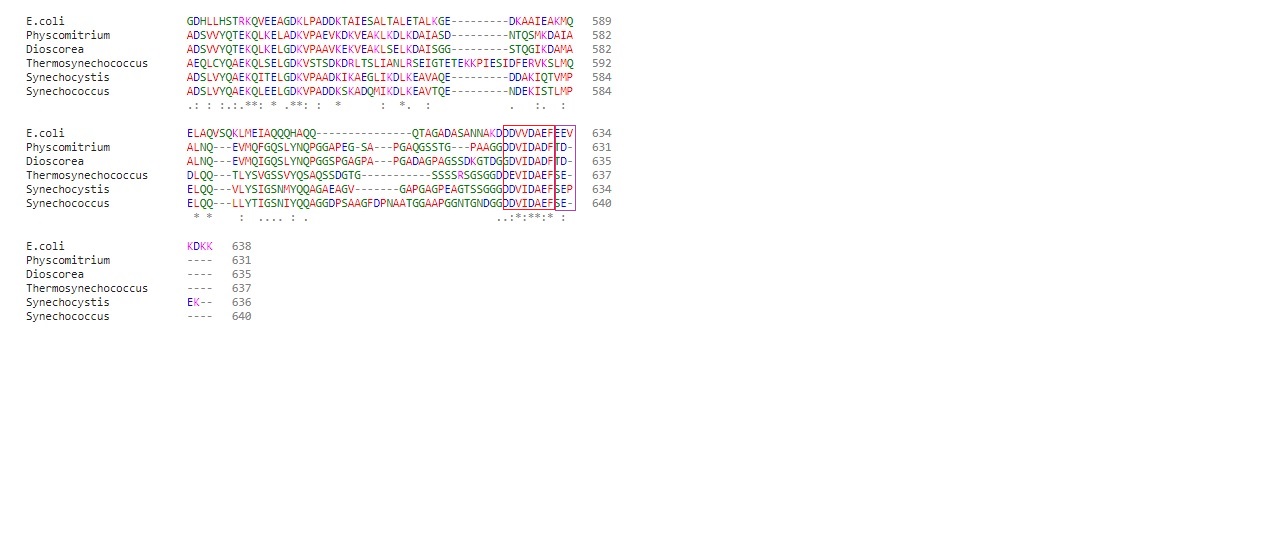
**

**Figure S2***.* Clustal omega alignment of whole amino acid sequence of *E.coli* DnaK and its six cyanobacterial homologues.

*
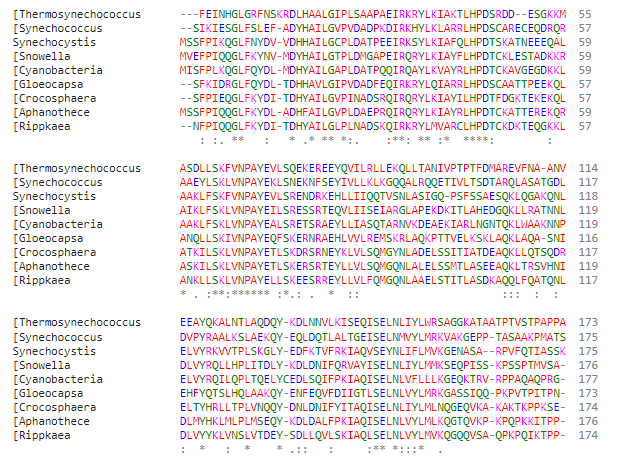
*

*
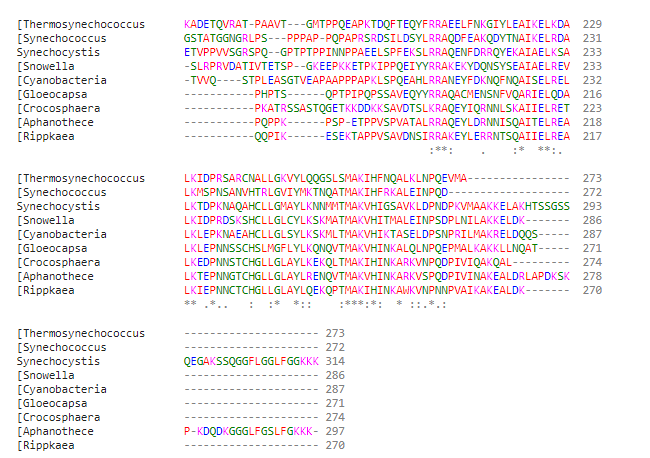
*

**Figure S3***.* Clustal omega alignment of whole sequences of DnaJ Sll1384 from different cyanobacteria species. TPR domain marked with a red frame.


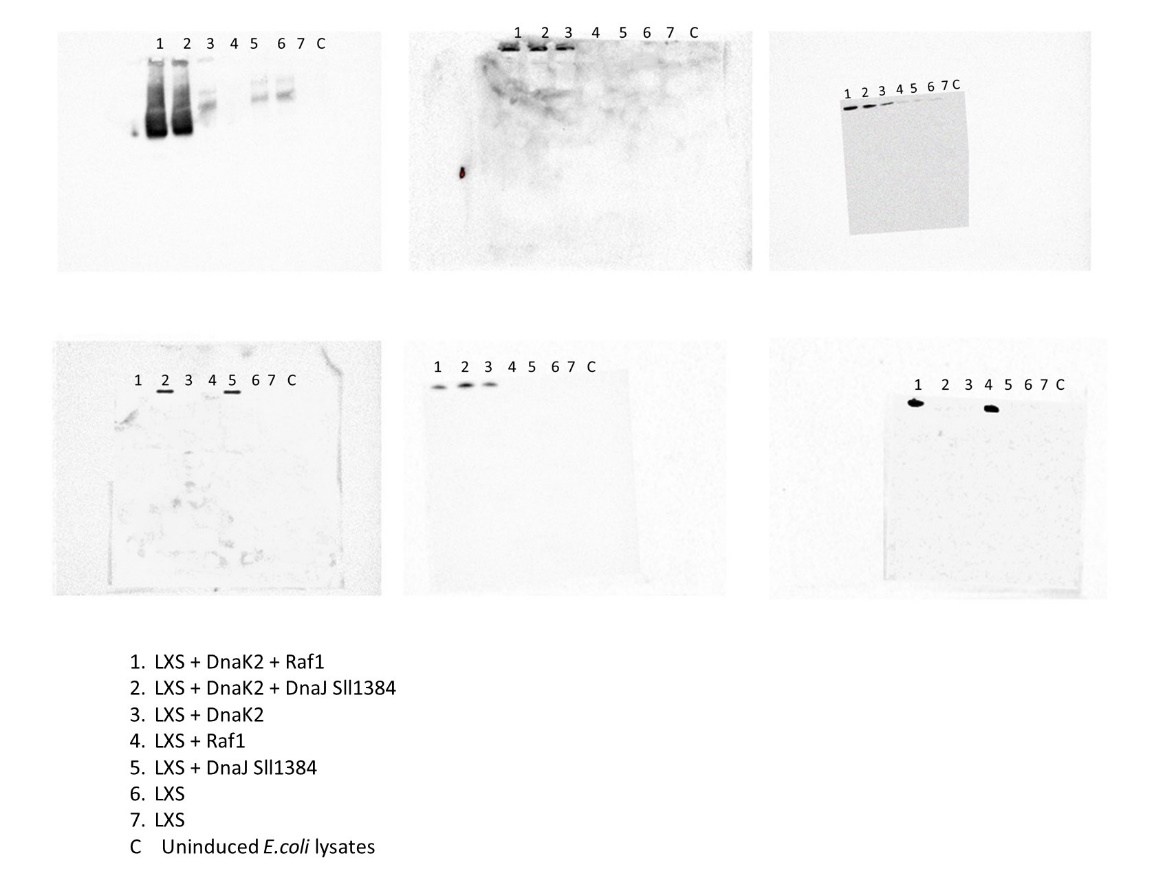


Figure S4. Full range blots data for Figure 4.

Table S1. Rate of fluorescence decrease, representing chaperone binding to RbcL peptide, measured by ANS displacement assay. DnaJ/DnaK (final concentration indicated) were added directly to RbcL-ANS mixture.

| **Protein** | **Slope** | **SD** ± |
| --- | --- | --- |
| **DnaJ Sll1384** |  |  |
| 3 uM | 1316,5 | 20,18 |
| 2uM | 87,3 | 11,2 |
| 1uM | 74,5 | 23,9 |
| **DnaK2** |  |  |
| 5 uM | 267,2 | 8,51 |

*
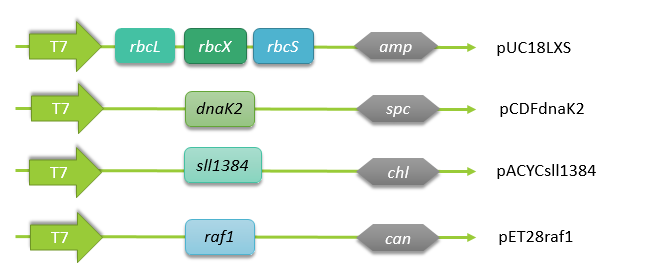
*

Figure S5. Cloning cassette in plasmids used for co-expression, with indicated protein gene and specific resistance (amp- ampicillin, spc- spectomycin, chl- chloramphenicol, kan- kanamycin). Names of plasmid given in each rows.


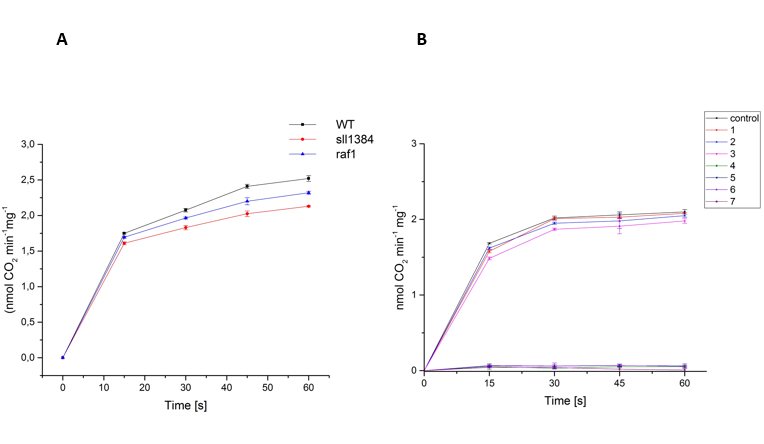


Figure S6. Kinetics of Rubisco activity. Panel A presents kinetics of Rubisco activity in cyanobacterial extracts (activity presented on the figure 5B in the manuscript), panel B presents kinetic of Rubisco activity presented on the figure 4B in the manuscript)


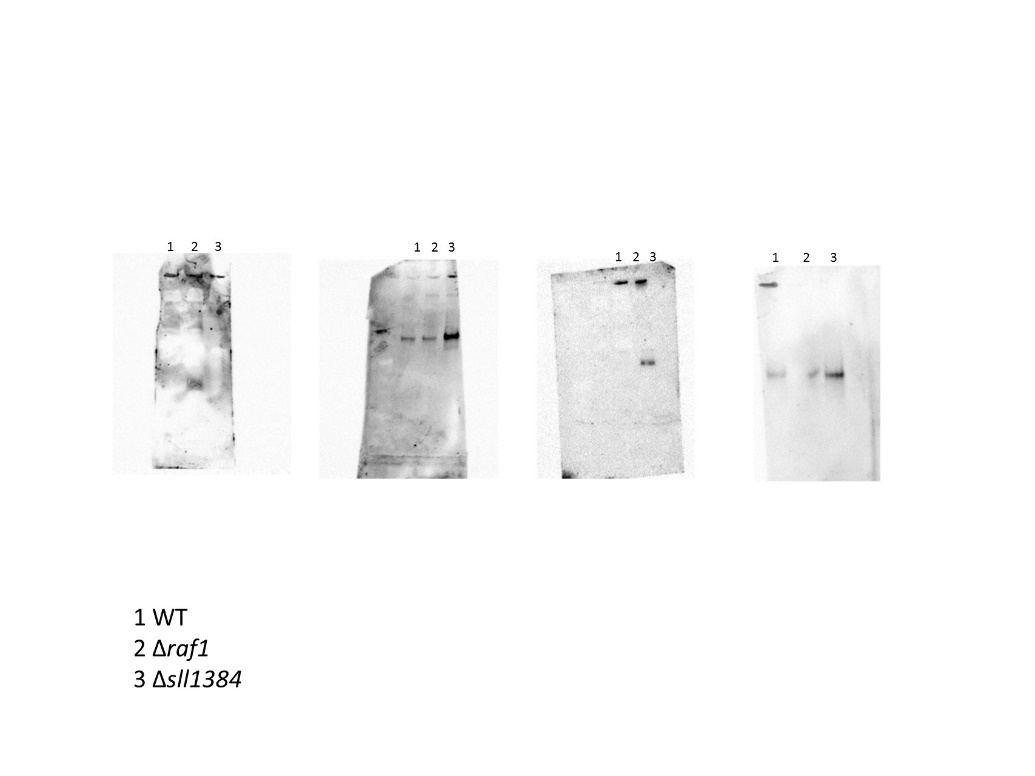


Figure S7. Full blots for figure 5.


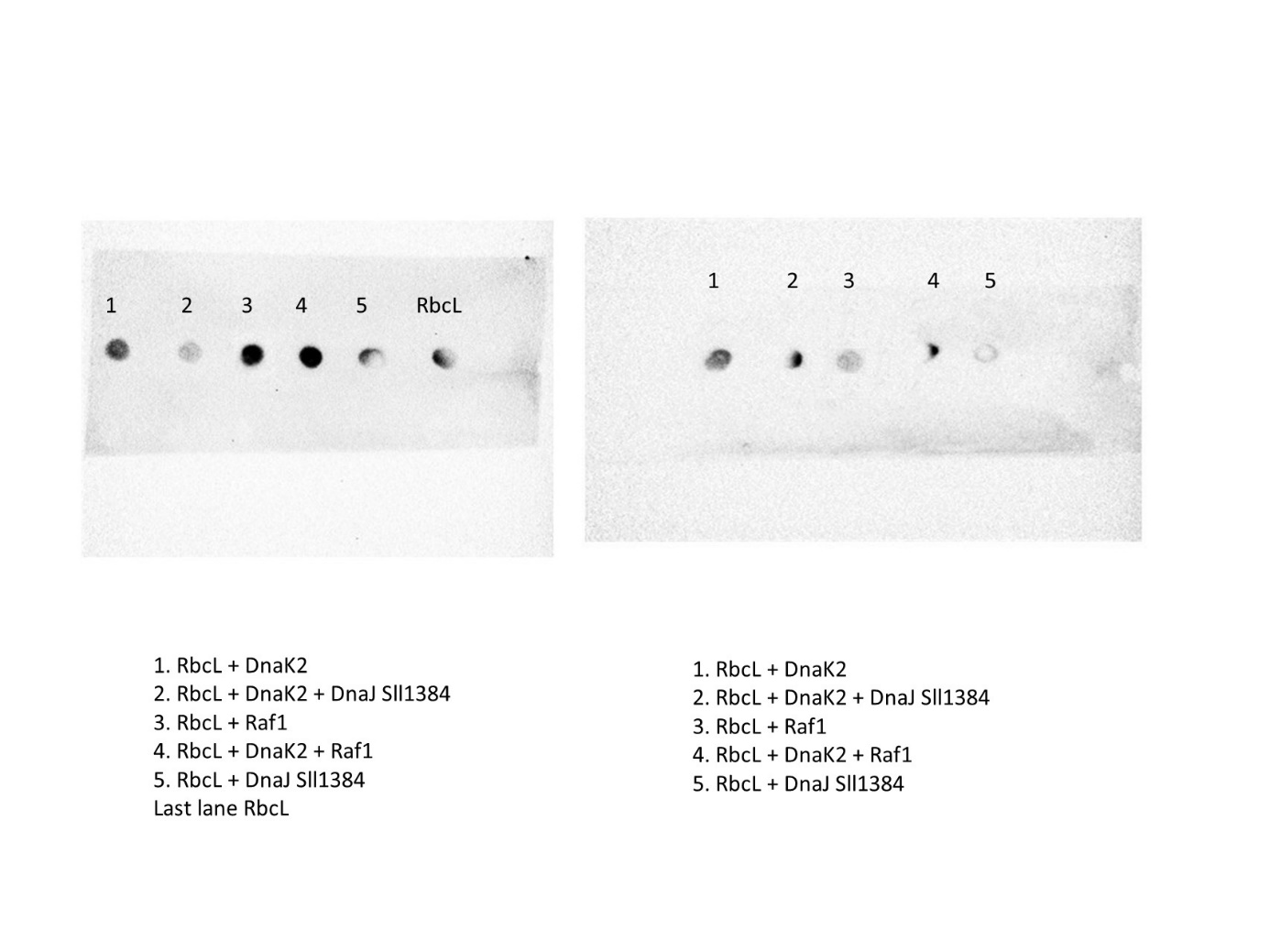


Figure S8. Full dot blots from figure 3.

Figure S9. Densitometry analysis of dot-blots of figure 3. (A) Total RbcL and soluble RbcL quantification, bars are the average of results obtained for two independent biological replicates, error bars are the maximal deviation for that probe. (B) the relative content of soluble RbCl in its total fraction. Sample coding (1-5) as on Fig. S8.
